# Protective Effects of Probiotics on Runners’ Mood: Immunometabolic Mechanisms Post-Exercise

**DOI:** 10.3390/nu16213761

**Published:** 2024-11-01

**Authors:** Edgar Tavares-Silva, Valdir de Aquino Lemos, Elias de França, Jean Silvestre, Samile Amorim dos Santos, Graziela Rosa Ravacci, Ronaldo Vagner Thomatieli-Santos

**Affiliations:** 1 Post-Graduate Program in Psychobiology, Federal University of São Paulo, São Paulo 04040-003, SP, Brazilaquino.lemos@unifesp.br (V.d.A.L.); 2 Department of Bioscience, Federal University of São Paulo, Rua Silva Jardim, 136, Vila Mathias, Santos 11015-021, SP, Brazil; elias.franca@unifesp.br (E.d.F.); jean.carlos@unifesp.br (J.S.); samile.amorin@gmail.com (S.A.d.S.); grazielametanutri@gmail.com (G.R.R.)

**Keywords:** strenuous exercise, fatigue, microbiota, inflammation, gut–brain axis, immunonutrition, mental health

## Abstract

Background: The gut–brain axis may mediate mood changes due to strenuous exercise. Therefore, probiotic supplementation may mitigate mood worsening. Purpose: The present study aims to evaluate the effect of probiotic supplementation on mood and immunometabolic parameters after a marathon. Materials and methods: Fourteen marathon runners were selected and divided into placebo and probiotic groups that were supplemented for 30 days. Before and after the marathon, mood (POMS) was assessed, and blood was collected for analysis of immunometabolic parameters. Statistical analysis was performed, and *p* < 0.05 was considered to determine statistically differences. Results: Tension decreased after the marathon in both groups. Vigor decreased only in the placebo group. Fatigue increased after the marathon in both groups. TMD increased after the marathon in placebo. The IL2/IL-4 ratio decreased in the probiotic group after the marathon compared to before and increased compared to the placebo group. The IL-10 increased after the marathon in placebo. TNF-α increased after the marathon in probiotics. The TNF-α/IL-10 ratio decreased after the marathon in both groups. LPS decreased in the probiotic group after the marathon compared to before and in the placebo group. Conclusions: Thirty days of probiotic supplementation attenuated the impact of marathons on mood worsening. The decrease in LPS in the probiotic group mediated the change in the pro/anti-inflammatory balance, indicating an immunometabolic mechanism by which the gut–brain axis impacts mood after strenuous exercise.

## 1. Introduction

Although exercise promotes health improvements, recent studies show that acute strenuous exercise, such as marathon running, impairs mood [1,2,3]. Several non-exclusive mechanisms are associated with worsening mood after strenuous exercise, including changes in serotonin and cortisol concentrations, dehydration, hypoglycemia, and neurophysiological changes [1,4]. In addition, elevation of pro-inflammatory cytokines, such as IL-6 and TNF-α, may also be associated with worsening mood after strenuous exercise [1].

Over the last 10 years, there has been growing interest in understanding the importance of the gut–brain axis for those who exercise. Increased blood inflammatory biomarkers influence glial cells residing in the central nervous system, as well as microglia, astrocytes, and oligodendrocytes, causing neuroinflammation and worsening mood [5,6]. This is an important communication pathway between the gut and the brain, especially during physical exercise. It has recently been proposed that exhaustive exercise can alter the composition of the intestinal microbiota, promoting dysbiosis that favors inflammation and producing negative consequences on homeostasis due to the interaction between the microbiota and different physiological tissues [7,8]. In this scenario, by rebalancing the intestinal microbiota, they can reduce bacterial translocation and improve the integrity of the intestinal barrier, limiting systemic inflammation and neuroinflammation.

However, more robust studies are needed to investigate the effects of probiotics on mood after marathon running. In this context, a recent hypothesis that dysbiosis of the intestinal microbiome may contribute to worsening mood [9] has gained prominence, and this suggestion may be even more vital for those who practice strenuous exercise. In this scenario, probiotic supplementation may be an efficient strategy to mitigate the undesirable impact of strenuous exercise such as marathon running on mood. Daily supplementation with *Lactobacillus* and *Bifidobacterium* improved mental health and metabolic parameters, especially insulin sensitivity, dyslipidemia, inflammation, and antioxidant capacity, and reduced health service utilization in healthy individuals [10]. A recent study showed that supplementation with the probiotics *Bifidobacterium lactis W51*, *Levilactobacillus brevis W63*, *Lactobacillus acidophilus W22*, *Bifidobacterium bifidum W23*, and *Lactococcus lactis W58* influences the composition of the intestinal microbiota and the permeability of epithelial cells, improving the inflammatory response in athletes [11]. However, different supplementation protocols with other strains and probiotics may result in different effects.

This study aims to evaluate whether 30 days of supplementation with *Lactobacillus acidophilus*, *Lactobacillus lactis*, *Bifidobacterium lactis*, and *Bifidobacterium bifidum* affects mood and inflammatory markers after a marathon. This study hypothesizes that probiotic supplementation may attenuate the impact of marathon running on mood by reducing the systemic inflammatory response and LPS after a marathon.

## 2. Materials and Methods

### 2.1. Ethical Aspects

This study was approved by the UNIFESP Research Ethics Committee (CEP (#691.519 2014) and respected the rules established by Brazilian law in Resolution No. 12 of the National Health Council. To participate in the study, the volunteers initially had all the necessary information, including the evaluations, and subsequently signed a free and enlightened consent (ICF) term.

### 2.2. Sample Size

Fourteen male people were part of the study and underwent an acute exercise session as described below. Inclusion criteria: being in training (performing aerobic exercise at least three times a week, for at least one year), being male, being between 30 and 45 years old, being eutrophic, and being able to run a marathon. People who had heart disease, infections, diabetes, obesity, hypertension, or any other chronic disease as assessed by a doctor, smokers, users of drugs of abuse, users of any medication (anti-inflammatories, corticosteroids, and antibiotics) that could interfere with the results of the study, frequent consumers of alcoholic beverages (more than three times a week), and users of any non-carbohydrate supplement during the period of the experiment were restored.

### 2.3. Study Design

The volunteers performed cardiovascular tests (rest and effort and ergospirometry electrocardiogram) and received supplementation. The division of the groups was randomized, and volunteers did not know which supplement they would ingest, as described below:

Control group—placebo—seven volunteers who consumed placebo supplementation of probiotics (2.0 g cornstarch per day) in gelatinous capsules handled in pharmacies, similar to the capsules consumed by the probiotic group regarding color, taste, smell, and size for 30 days, ingested at night. Probiotic group—seven volunteers who consumed supplementation of 2.0 g per day of probiotics in gelatinous capsules handled in pharmacies containing *Lactobacillus acidophilus*, *Lactobacillus lactis*, *bifidobacterium lactis*, and *bifidobacterium bifidum* 10^9^ × CFU in each bacteria strain per day, for 30 days, ingested in the evening. Volunteers from both groups were self-reporting supplementation not causing verified disorders due to the components. In the first visit after the signing of the free and informed consent form, body composition was assessed to characterize the sample, a clinical evaluation was made, and medical authorization was given for the practice of physical exercise. At the end of the first visit, the volunteer supplement was delivered. During the supplementation period, to identify the dietary profile, the volunteers answered a food registration questionnaire. After thirty days of supplementation, the strenuous physical exercise protocol (marathon) was performed. In this second moment, blood was collected, and the questionnaires were answered again. During the marathon, the volunteers consumed a carbohydrate drink with a concentration of 6% at km 18, 29, 31, and 36. The volunteers were allowed to drink water *ad libitum* throughout the race.

### 2.4. Body Composition

Body mass was measured on a scale with an accuracy of 0.1 g. Height was measured using a vertical stadiometer with an accuracy of 1 mm. Body composition was determined by plethysmography following the calibrations and standardizations recommended by the manufacturer (COSMED USA Inc., Illinois, 2211 North Elston Avenue Suite 305, Chicago, IL, USA).

### 2.5. Determination of VO_2peak_

VO_2peak_ was determined by running on a treadmill at an initial speed of 7.0 km/h for three minutes and increasing the load by 1.0 km/h per minute until voluntary exhaustion. Two criteria were used to characterize exhaustion and test interruption: difficulty in sustaining the speed for 15 s or the participant’s request to stop even after being encouraged to continue. The test was performed at a 1% treadmill incline. Respiratory variables were analyzed using a COSMED^®^ Quark PFT—Pulmonary Function Testing—FRC & DLCO gas analyzer (COSMED USA Inc., Illinois, 2211 North Elston Avenue Suite 305, Chicago, IL, USA). Temperature and humidity were controlled at 22 °C and 61%, respectively.

### 2.6. Blood Collection

Blood samples were collected at 2 moments: before and immediately after the marathon. Volunteers remained in a tent set up at the marathon site, which was designated for blood collection and rest, both before and after the marathon. Adequate water, chairs, and mats for stretching were provided. At each of the 2 points, 25 mL of blood was drawn from the antecubital vein. Following collection, the blood samples were centrifuged at 690× *g* for 15 min at 4 °C until plasma or serum was extracted. The samples were kept in a freezer at 80 °C until analysis.

### 2.7. Blood Measurements

Serum concentrations of IL-2, IL-4, IL-6, IL-10, and TNF-α were determined by multiplex using Merck/Millipore^®^ kits (code HCYTA-60K) and Luminex technology with magnetic beads. A Luminex 200™ analyzer with Magpix^®^ system and Milliplex^®^ Analyst 5.1 software was used. LPS concentration was determined by ELISA using MyBiosource^®^ kits (code MBS702450). Glucose and glutamine concentrations were determined enzymatically using, respectively, Bioclin^®^ (code K082-3) and Sigma^®^ (code GLN1-1KT) kits. Serotonin concentration was measured by ELISA using the ALPCO^®^ kit (code 17-SERHU-E01-FST). All analyses were performed according to the manufacturer’s protocol and standards.

### 2.8. Assessment of Mood

The Brunel Mood Scale was developed to subjectively measure mood [12]. The scale was adapted from the Profile of Mood [5] and validated for the Brazilian population by Rohlfs et al. [13]. It consists of a list of 24 mood-related adjectives rated on a Likert-type scale in which 0 means “not at all” and 4 means “strongly”. Higher values indicate a more robust endorsement of tension, depressed mood, anger, fatigue, confusion, and total mood disorder, while higher values indicate worsening vigor.

### 2.9. Eating Pattern

A 3-day food record was used to determine the eating pattern. To do this, the volunteers answered a questionnaire two days in a row and one day on the weekend. The Webdiet^®^ software processed the information collected and indicated the usual eating patterns of the volunteers at the beginning of the study.

### 2.10. Statistical Analysis

Data are expressed as mean ± standard deviation, and statistical tests were applied according to data normality, which was verified using the Shapiro–Wilk test. Repeated measures analysis was performed using a two-way ANOVA test or Student’s *t*-test, with Fisher’s LSD post-hoc. The significant level adopted was *p* ≤ 5%. IBM SPSS Statistics 20 software was used to perform the analyses. We conducted a posteriori sample size calculation with G*Power 3.1 so as not to assume type 1 or 2 errors. Thus, regarding the two-way ANOVA test, the parameters were within–between interaction, two groups, two measurements (before and after the marathon), 14 participants, alpha error of 5%, and power of 0.80 (probability of rejecting a null hypothesis), and the minimum effect size of comparisons was 0.38.

## 3. Results

**Figure 1:**
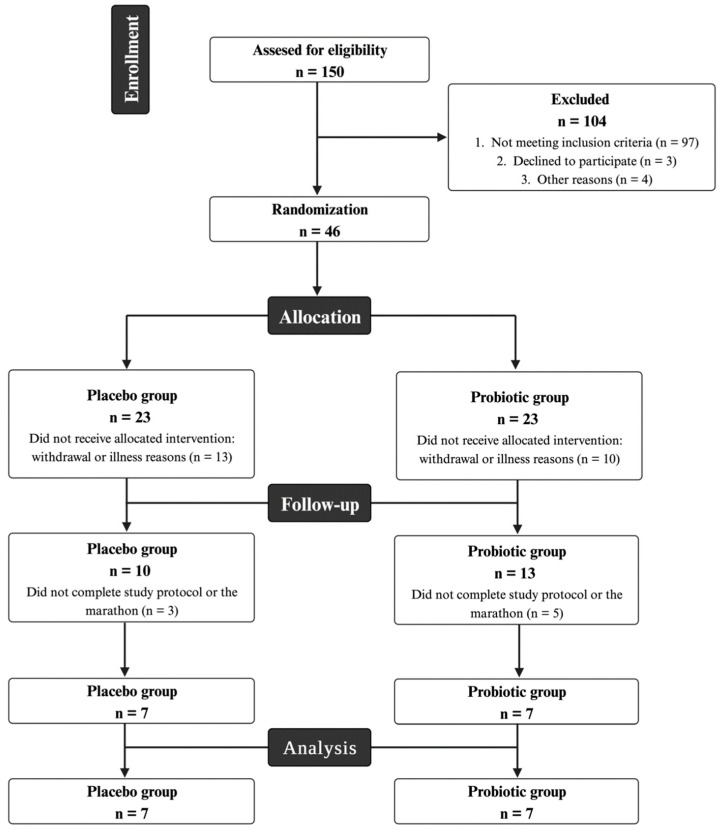
Participation flowchart.

One hundred and fifty runners were assessed for eligibility; however, only 46 were eligible for the study and randomized to the placebo or probiotic group. After randomization, some participants were excluded for health reasons, and others withdrew from the study. We allocated 10 participants in the placebo group and 13 in the probiotic group. Runners who did not complete the dietary supplementation protocol or did not complete the marathon were excluded from the final analysis. Finally, the present study sample comprised 14 male runners, with seven runners in each group. The participation flowchart is illustrated in Figure 1. The characteristics of the participants were not different between groups at the beginning of the supplementation period, as shown in Table 1, including anthropometric, body composition, nutritional, and health characteristics.

The mood results are presented in Table 2. Tension was lower after the marathon than before in both groups (*p* = 0.05). Vigor was lower after the marathon in the placebo group (*p* = 0.05). Fatigue was higher after the marathon in both groups compared to pre-marathon values (*p* = 0.5). TMD was higher after the marathon than before in the placebo group (*p* = 0.05). There were no differences in anger, depression, and confusion in relation to time points and groups.

Table 3 shows the results of the plasma parameters. The IL2/IL-4 ratio was lower in the probiotic group after the marathon compared to the value before the marathon (*p* = 0.01). However, it was higher in the probiotic group compared to the placebo group after the marathon (*p* < 0.02). The IL-10 concentration was higher after the marathon in the placebo group compared to before the marathon (*p* = 0.02). The TNF-α concentration was higher after the marathon than before in the probiotic group (*p* = 0.01). The TNF-α/IL-10 ratio was lower after the marathon in both groups compared to the values before the marathon (*p* = 0.004 for the placebo group and *p* = 0.001 for the probiotic group). The LPS concentration was higher after the marathon compared to before the marathon and compared to the placebo group after the marathon (*p* = 0.05). There was no difference in the concentrations of IL-2, IL-4, IL-6, glucose, serotonin, and glutamine.

## 4. Discussion

The study aimed to evaluate the effect of 30 days of supplementation with a combination of probiotics on mood and immunometabolic parameters after a marathon. This was a double-blind, placebo-randomized study. The main findings show that supplementation was able to mitigate the worsening of mood, notably by preserving vigor and TMD after the marathon, accompanied by an improvement in the pro/anti-inflammatory balance indicated by the IL-2/IL/4 ratio and by a reduction in LPS after the marathon. These results deserve to be highlighted for their novelty, including the combination of probiotics, the time of supplementation, the population studied, and the context of the study, which enhances our understanding of the relationship between the gut and the brain.

The results indicate that there was no difference between the participants at the beginning of the study and that the groups were homogeneous. This is an essential condition for the study since several factors can influence the response to probiotic supplementation, such as body composition and age [8]. In addition, we observed that there was no significant difference between the time taken to complete the marathon and the VO_2peak_. This information together confirms that the physical conditioning of the participants in both groups was similar, which also brings relevance to the study because physical conditioning and training levels can impact the composition of the microbiota and the response to probiotic supplementation [14].

Recent studies have focused on the impact of probiotic supplementation on aspects of the immune response [15,16,17]. Other studies have shown a relationship between the composition of the intestinal microbiota and different sports modalities, physical conditioning, and athletic performance [14].

However, the scientific literature still lacks substantial information regarding the impact of the gut–brain axis on psychobiological relationships during and after strenuous physical exercise in trained individuals. A website specializing in marathon running estimates that there are 11 million marathon runners in the world, representing 0.15% of the world population, who in total run approximately 4000 marathons per year [18]. Added to this percentage is an undefined number of people who train regularly, even with strenuous exercise overloads, but who do not compete in marathons. Therefore, the findings of this study may have a broad impact on people who exercise regularly.

Mood is an essential state for the mental health and well-being of people who practice physical exercise, and mood changes can affect performance [19]. Preserving mental balance during strenuous exercise such as marathon running is essential for performance. However, several studies have shown deterioration during and after marathons of several dimensions that make up mood [20].

In the present study, there was a change in TMD after the marathon, which indicates a worsening of mood in marathon runners and confirms previous studies of strenuous exercise [21,22]. In fact, TMD is a broad indicator of mood since it is calculated by adding the different dimensions that make up the mood state, which are tension, depression, anger, and fatigue, and subtracting the vigor dimension from this sum. On the other hand, there was no statistical difference in DMR after the marathon in the group supplemented with a probiotic combination consisting of 1 × 10^9^ CFU of *Lactobacillus acidophilus*, *Lactobacillus lactis*, *bifidobacterium lactis*, and *bifidobacterium bifidum*.

This result demonstrates that the supplementation strategy employed in this study was able to mitigate the damage caused by the marathon on mood. The worsening in TMD in the placebo group was due to the decrease in tension and vigor and an increase in fatigue after the marathon, as expected. The 30 days of probiotic supplementation used in this study were able to maintain vigor after the marathon, which ensured the preservation of TMD in the probiotic group. Interestingly, in a recent study, badminton players received a commercial probiotic drink containing *Lactobacillus Casei* at a dose of 3 × 10^10^ CFU daily for six weeks and showed no change in mood after a battery of exercises [23]. Other previous studies also did not observe the effects of different probiotic supplementation protocols on mood under stress conditions [24,25].

Comparison of the results of the present study with the literature and the divergences in results indicate that the effects of probiotic supplementation are dependent on several factors, including the time of supplementation, the population studied, and the combination of probiotics. Thus, the results found in the present study cannot be extrapolated to other populations and different contexts since the mechanisms by which the different supplementation protocols may act have yet to be fully known.

Our focus from then on was directed to observe the possible immunometabolic influences on the regulation of mood in marathon runners after 30 days of probiotic supplementation. Several studies have shown that inflammation, changes in serum cytokine and LPS concentrations, and even neuroinflammation can contribute to mood deterioration and worsening of other conditions [26,27,28,29].

Strenuous exercise, including marathon running, promotes changes in the production and concentration of several cytokines in the bloodstream [30,31,32]. At the same time, recent studies have observed deterioration of the intestinal microbiota and the barrier function of the gastrointestinal tract after strenuous exercise, which results in increased intestinal permeability and circulating LPS [33,34].

Our results partially confirm the literature, as we found an increase in IL-10 concentration after marathon running in the placebo group, which resulted in a reduction in the TNF-α/IL-10 ratio and an improvement in the pro/anti-inflammatory balance, in contrast to previous studies that recorded an increase in the inflammatory profile [30,31]. Several factors can influence cytokine concentration during and after strenuous exercise, such as physical conditioning, exercise intensity, and the nutritional status of the runners [35]. The researchers instructed the runners to consume water and carbohydrates during the marathon following the supplementation and hydration that each runner was used to, including carbohydrate drinks with a concentration of 6% at km 18, 29, 31, and 36 and water *ad limit*. Thus, there was no difference in blood glucose in both groups after the marathon, which suggests that the changes in cytokine concentration found in this study were not due to changes in the availability of glucose and muscle glycogen [36].

Probiotic supplementation influenced the pro/anti-inflammatory balance towards an anti-inflammatory pattern, confirmed by the decrease in the IL-2/IL-4 and TNF-α/IL-10 ratios despite the increase in TNF-α. Previous studies have also evaluated the concentration of cytokines in the bloodstream after probiotic supplementation; in this sense, our results do not confirm previous studies [8]. Once again, we suggest that the divergences in the literature are due to the different supplementation protocols used in the studies. Classical studies have successfully shown that peripheral cytokines can cross the blood–brain barrier through specific mechanisms and thus control the balance of neurotransmitters such as serotonin and the activity of the hypothalamic–pituitary–adrenal axis and microglia, mechanisms by which acute and chronic inflammation influence mood [37].

Furthermore, probiotic supplementation reduced LPS concentration after running the marathon. This result confirms previous studies that also found a reduction in LPS after strenuous exercise due to probiotic supplementation [38] and indicates the potential of the supplementation used in the present study to preserve the barrier function in the intestinal epithelium and regulate the gut–brain axis through immunometabolic pathways.

In fact, studies show that LPS increases BBB permeability, allowing peripheral metabolites and inflammatory cytokines to cross the barrier and induce CNS dysfunctions [39,40,41]. In addition, LPS is also able to cross the B and binding to TLR-4 receptors, activating the NFKβ pathway in microglia, resulting in neuroinflammation [42]. Fatigue can be induced by these pro-inflammatory molecules entering the brain or by LPS-mediated neuroinflammation, impairing serotonergic neurons, the hypothalamic–pituitary–adrenal axis, and the basal ganglia, leading to behavioral symptoms associated with fatigue [43,44,45].

Despite the interesting results, this study has some limitations, including the fact that there were no women in the sample. Furthermore, there are doubts as to whether these results can be extrapolated to people who practice other types of exercise. Therefore, the results of this study should be viewed with caution, and further studies should be conducted with a more significant number of participants who perform other types of exercise, including both men and women in the sample. In addition, the supplementation with *Lactobacillus acidophilus*, *Lactobacillus lactis*, *bifidobacterium lactis*, and *bifidobacterium bifidum*, which was used in this study, may not have the same effects in other populations or other outcomes.

## 5. Conclusions

The set of results presented and the discussion lead us to the conclusion that 30 days of probiotic supplementation composed of *Lactobacillus acidophilus*, *Lactobacillus lactis*, *bifidobacterium lactis*, and *bifidobacterium bifidum* was able to attenuate the impact of marathon running on mood worsening, especially preserving vigor and TMD. The decrease in LPS concentration in the probiotic-supplemented group mediated the change in the pro/anti-inflammatory balance, indicating a possible immunometabolic mechanism by which the gut–brain axis impacts mood regulation after strenuous exercise.

## Figures and Tables

**Table 1:** Descriptive characteristics of participants randomized to probiotic and placebo at baseline.

Variable	Placebo	Probiotic	*p*-Value
Age (years)	38.28 ± 3.09	41.57 ± 3.20	0.075
Body mass (kg)	78.43 ± 8.40	71.24 ± 3.55	0.059
Height (cm)	179.36 ± 5.23	175.82 ± 3.01	0.14
BMI (kg/m^2^)	24.90 ± 1.81	23.08 ± 1.83	0.08
Fat mass (kg)	16.03 ± 6.29	10.95 ± 2.29	0.06
Fat-free mass (kg)	64.47 ± 8.49	60.77 ± 4.27	0.32
Marathon time (min)	243.0 ± 33.73	252.87 ± 39.77	0.62
Average Speed (km/h)	10.73 ± 1.53	10.41 ± 1.48	0.70
VO_2Peak_ (kg/mL/min)	54.53 ± 6.88	56.92 ± 8.35	0.57
Maximum HR (Bpm)	182.16 ± 10.05	178.70 ± 3.45	0.40
TCV (kcal)	1994.46 ± 365.73	2434.69 ± 505.53	0.08
Carbohydrate (%)	47.77 ± 4.24	47.88 ± 16.98	0.98
Carbohydrate (g)	237.46 ± 61.11	294.52 ± 122.76	0.29
Proteins (%)	18.92 ± 1.62	17.53 ± 4.07	0.42
Proteins (g)	92.66 ± 7.10	105.95 ±38.37	0.38
Lipids (%)	33.28 ± 2.76	34.56 ± 12.96	0.80
Lipids (g)	74.88 ± 10.65	92.52 ± 51.14	0.38
Men (*n*)	7	7	-
Women (*n*)	0	0	-
Smokers (*n*)	0	0	-
Obese (*n*)	0	0	-
Diabetics (*n*)	0	0	-
Hypertensive (*n*)	0	0	-
Other chronic diseases (*n*)	0	0	-

Data presented as mean ± standard deviation. The comparison between groups was performed using Student’s *t*-test. Caption: BMI—body mass index, and TCV—total calorie value. *n* = 7 marathon runners in each group.

**Table 2:** The mood of runners from placebo and probiotic groups at baseline and after the marathon.

Variable	Placebo Group		Probiotic Group	
	Before	After	Before	After
Tension	5.17 ± 4.45	0.66 ± 1.03 ^a^	4.57 ± 3.74	0.71 ± 1.11 ^a^
Vigor	12.2 ± 2.71	5.50 ± 2.88 ^a^	11.0 ± 4.73	8.14 ± 4.06
Anger	0.00 ± 0.00	0.33 ± 0.51	0.28 ± 0.75	0.28 ± 0.48
Depression	0.00 ± 0.00	0.16 ± 0.40	0.14 ± 0.37	0.85 ± 1.21
Fatigue	1.17 ± 2.40	9.00 ± 3.10 ^a^	2.71 ± 3.73	8.43 ± 4.43 ^a^
Confusion	1.00 ± 0.89	0.16 ± 0.40	0.71 ± 1.50	1.57 ± 3.05
TMD	−4.83 ± 7.28	4.83 ± 4.67 ^a^	−2.57 ± 5.86	3.71 ± 10.00

TMD: Total mood disturbance. Results are expressed as the mean ± (standard deviation). Variables were compared using two-way ANOVA and Fisher’s LSD post-hoc. ^a^ different in relation to before. *n* = 7 marathon runners in each group and *p* < 0.05.

**Table 3:** Serum parameters in placebo and probiotic groups at baseline and after the marathon.

	Placebo		Probiotic	
	Before	After	Before	After
IL-2 (pg/mL)	0.53 ± 0.11	0.53 ± 0.10	3.36 ± 3.0	2.38 ± 2.0
IL-4 (pg/mL)	1.07 ± 0.26	1.65 ± 0.50	1.16 ± 0.70	2.12 ± 1.3
IL-2/IL-4	0.54 ± 0.05	0.47 ± 0.12	1.15 ±0.5	0.76 ± 0.16 ^ab^
IL-10 (pg/mL)	0.47 ± 0.09	28.83 ± 18.97 ^a^	0.36 ± 0.10	10.20 ± 7.70
TNF-α (pg/mL)	2.06 ± 0.16	3.33 ± 0.65	2.48 ± 0.80	4.31 ± 1.2 ^a^
TNF-α/IL-10	5.07 ± 0.7	1.53 ± 0.49 ^a^	6.58 ± 1.71	2.66 ± 1.02 ^a^
IL-6 (pg/mL)	7.42 ± 4.53	7.74 ± 5.02	19.67 ± 10.0	17.45 ± 9.80
LPS (pg/mL)	50.8 ± 31.6	52.0 ± 20.4	51.3 ± 20.8	29.9 ± 13.9 ^ab^
Glucose (mg/dl)	124.33 ± 33.84	150.38 ± 42.55	145.99 ± 20.18	152.67 ± 29.99
Glutamine (nmol/mL)	52.55 ± 22.6	33.50 ± 5.90	41.16 ± 16.70	37.70 ± 11.70
Serotonin (ng/mL)	331.47 ± 94.88	286.66 ± 101.47	395.72 ± 86.71	381.14 ± 93.07

Results showed as the mean ± (standard deviation). Cytokines and LPS are expressed in pg/mL, glucose in mg/dl, glutamine in nmol/mL, and serotonin in ng/mL. Variables were compared using two-way ANOVA and Fisher’s LSD post-hoc. ^a^ Different in relation to before, and ^b^ different in relation to placebo. *n* = 7 marathon runners in each group and *p* < 0.05.

## Data Availability

Not applicable.
